# Antidiabetic Effects of Chamomile Flowers Extract in Obese Mice through Transcriptional Stimulation of Nutrient Sensors of the Peroxisome Proliferator-Activated Receptor (PPAR) Family

**DOI:** 10.1371/journal.pone.0080335

**Published:** 2013-11-12

**Authors:** Christopher Weidner, Sylvia J. Wowro, Morten Rousseau, Anja Freiwald, Vitam Kodelja, Heba Abdel-Aziz, Olaf Kelber, Sascha Sauer

**Affiliations:** 1 Otto Warburg Laboratory, Max Planck Institute for Molecular Genetics, Berlin, Germany; 2 Department of Biology, Chemistry, and Pharmacy, Free University of Berlin, Berlin, Germany; 3 Scientific Department, Steigerwald Arzneimittelwerk GmbH, Darmstadt, Germany; Wageningen University, Netherlands

## Abstract

Given the significant increases in the incidence of metabolic diseases, efficient strategies for preventing and treating of these common disorders are urgently needed. This includes the development of phytopharmaceutical products or functional foods to prevent or cure metabolic diseases. Plant extracts from edible biomaterial provide a potential resource of structurally diverse molecules that can synergistically interfere with complex disorders. In this study we describe the safe application of ethanolic chamomile (*Matricaria recutita*) flowers extract (CFE) for the treatment and prevention of type 2 diabetes and associated disorders. We show in vitro that this extract activates in particular nuclear receptor peroxisome proliferator-activated receptor gamma (PPARγ) and its isotypes. In a cellular context, in human primary adipocytes CFE administration (300 µg/ml) led to specific expression of target genes of PPARγ, whereas in human hepatocytes CFE-induced we detected expression changes of genes that were regulated by PPARα. In vivo treatment of insulin-resistant high-fat diet (HFD)-fed C57BL/6 mice with CFE (200 mg/kg/d) for 6 weeks considerably reduced insulin resistance, glucose intolerance, plasma triacylglycerol, non-esterified fatty acids (NEFA) and LDL/VLDL cholesterol. Co-feeding of lean C57BL/6 mice a HFD with 200 mg/kg/d CFE for 20 weeks showed effective prevention of fatty liver formation and hepatic inflammation, indicating additionally hepatoprotective effects of the extract. Moreover, CFE treatment did not reveal side effects, which have otherwise been associated with strong synthetic PPAR-targeting molecules, such as weight gain, liver disorders, hemodilution or bone cell turnover. Taken together, modulation of PPARs and other factors by chamomile flowers extract has the potential to prevent or treat type 2 diabetes and related disorders.

## Introduction

Metabolic disorders such as type II diabetes and obesity show strikingly increasing global incidences, with more than 200 million people suffering from diabetes and more than 1 billion overweight or obese people worldwide [1]. These common diseases are not restricted to Western societies, but cumulatively affect people in newly industrializing countries [2]. In addition to associated comorbidities such as atherosclerosis, dyslipidemia, and cancer, metabolic disorders have a huge impact on the quality of life and the public health systems, and require novel approaches for effective prevention and treatment. Dietary and lifestyle interventions may efficiently counteract the cumulative development of metabolic disturbances, for example by early complementation with safe preventive phytopharmaceuticals or application of tailored food supplements [3].

Peroxisome proliferator-activated receptors (PPARs) are a family of nuclear receptors that can be activated by binding of various natural molecules including a number of dietary small molecules [4]. The gene-regulating PPARs play a central role in differentiation and glucose and lipid homeostasis, and can suppress inflammatory processes [5]. Pharmacologic modulation of PPARs is a common strategy to treat insulin resistance and dyslipidemia [5]. Three different subtypes of PPAR exist, PPARα, PPARβ/δ and PPARγ. These nuclear receptors are expressed in many different cell types [6]. PPARα, predominantly expressed in liver, controls fatty acid oxidation and lipoprotein metabolism, and is further involved in gluconeogenesis and ketone body biosynthesis. PPARβ/δ is ubiquitously expressed and has a central role in fatty acid oxidation and adaptive thermogenesis. PPARγ is highly expressed in adipocytes, but further controls differentiation and metabolic processes in liver, macrophages, bone cells and skeletal muscle. PPARγ is a key player in adipose tissue differentiation and maintenance by regulating energy storage and balance. This nuclear receptor is involved in glucose metabolism by regulating insulin sensitivity and represents a potent target for the treatment of diabetes [7]. Due to its large, hydrophobic binding pocket [4], PPARγ has been shown to mediate effects of dietary lipids and related small molecules. In general, to maintain cell homeostasis and physiology the PPARs play various, in part synergistic roles as environmental and nutrient sensors [8]. However, the activation of PPARγ requires decent fine-tuning to avoid potential side-effects as are for example known from synthetic antidiabetic compounds such as the thiazolidinediones. Due to strong but unspecific activation of PPARγ-directed gene expression in various tissues the thiazolidinediones induced liver diseases, weight gain, oedema, heart failure, osteoporosis, and cancer [9,10]. Selective PPAR modulators (SPPARMs) that maintain the antidiabetic potential of full PPARγ agonists but minimise potential side effects are promising for developing antidiabetic and antihyperlipidemic molecules with acceptable safety profiles [11-13]. Concerted activation of two or all three mentioned PPAR subtypes has further been proposed as an alternative, synergistic therapeutic approach [14].

Plant extracts provide an interesting resource for prevention and therapy of diverse diseases. The development of most established drugs is historically based on the identification of phytopharmaceuticals [15]. In addition, whole extracts or subfractions of various biomaterials are still a major medicinal application worldwide (e.g. by Traditional Chinese Medicine) [16], and have become a major focus of nutritional research to develop functional food and nutraceuticals with health benefits and good safety profiles [17]. 

Here we report the identification of ethanolic chamomile (*Matricaria recutita*) flowers extract as a phytopharmaceutical mixture to activate PPARγ resulting in considerable therapeutic effects on type 2 diabetes and dyslipidemia in insulin-resistant high-fat diet (HFD)-fed mice. The chamomile flowers extract further showed potent prevention of associated fatty liver disease without adverse side effects of classical PPAR agonists.

## Materials and Methods

Compounds were purchased from the following sources: rosiglitazone (Cayman Chemical, Ann Arbor, MI, USA), GW7647, GW0742 (Sigma-Aldrich, Taufkirchen, Germany). The chamomile flowers extract (CFE) used is a standardized [18] hydroethanolic (30% v/v) extract, which was provided by Steigerwald Arzneimittelwerk GmbH (Darmstadt, Germany) as a lyophilised powder. Main substance classes and approximative number of secondary phytochemical compounds for the CFE have recently been described [19].

### PPAR binding and transcriptional activation assays

Initially, we analysed the nine plant extracts of the widely used product Iberogast (STW5) [19]. CFE turned out to be a very efficient in activating PPARs as detected by time-resolved fluorescence resonance energy transfer (TR-FRET) - based assays according to the manufacturer’s protocol (Lanthascreen PPAR alpha/gamma/delta Competitive Binding Assay Kits, Life Technologies, CA, USA) as described recently [20]. Transcriptional activation of PPARs was assessed in cellular reporter gene assays according to the manufacturer’s protocols (GeneBLAzer PPAR alpha/gamma/delta Assay, Life Technologies). Briefly, HEK 293 cells were stably expressing a GAL4-PPAR-LBD fusion protein and an UAS-beta-lactamase reporter gene. Cells were incubated with different concentrations of compounds resulting in differential expression of the reporter gene. Binding and activation assays were measured with the POLARstar Omega (BMG LABTECH, Offenburg, Germany). Data were fitted using GraphPad Prism 5.0 according equation: Y=Bottom + (Top-Bottom)/(1+10^((LogEC_50_-X)*HillSlope)) with variable Hill slope.

### Cell culture

Primary subcutaneous preadipocytes (Zen-Bio, Research Triangle Park, NC, USA) were differentiated as described recently [20]. Adipocytes were treated for 24 h with 300 µg/ml chamomile flowers extract (CFE), 10 µM rosiglitazone (RGZ) or vehicle only. Human HepG2 cells (ATCC) were cultivated in DMEM (Gibco, Life Technologies) supplemented with 10% FBS and were treated for 24 h with 300 µg/ml chamomile flowers extract (CFE), 10 µM GW7647 or vehicle only.

Viability was assessed in HepG2 cells treated with CFE for 24 h using the CellTiter-Glo Luminescent Cell Viability Assay (Promega, Mannheim, Germany) according to the manufacturer’s protocol.

### PPAR knockdown

Specificity of PPARγ-driven modulation of gene expression in adipocytes was investigated using siRNA-mediated knockdown and subsequent real-time PCR detection. Therefore, differentiated human adipocytes were seeded in 24-well-plates (Nunc, Thermo Fisher Scientific, Langenselbold, Germany) at a confluence of 30 to 60%. Cells were transfected with 10 nM PPARγ Silencer Select siRNA (ID s10888) or 10 nM Silencer Select Negative Control 1 siRNA (all Life Technologies) using DeliverX Plus siRNA Transfection Kit (Panomics, Vignate-Milano, Italy). Transfection was carried out in serum- and antibiotic-free AM-1 medium (AM-1-PRF-SF, Zen-Bio) for 4 h and continued for 3 days in standard AM-1 medium. Afterwards, cells were treated with 300 µg/ml CFE, 10 µM RGZ or vehicle control for 24 hours prior to RNA collection.

Specificity of PPARα-driven modulation was investigated in siRNA-mediated knockdown in human hepatocytes and subsequent real-time PCR detection. Therefore, HepG2 cells were seeded in 24-well-plates (Nunc) at a density of 40,000 cells per well. Cells were transfected with 30 nM PPARα Silencer Select siRNA (ID s10880) or 30 nM Silencer Select Negative Control 1 siRNA (all Life Technologies) using the TransIT-TKO Transfection Reagent (Mirus Bio, WI, USA) according to the manufacturer’s protocol. Transfection was performed for 2 days, and cells were then treated with 300 µg/ml CFE, 10 µM GW7647 or vehicle control for additional 24 hours prior to RNA collection.

### RNA purification, cDNA synthesis and quantitative real-time PCR

Animal tissues (pool of 2 mice/group) were first lysed and homogenized in TRIzol (Life Technologies) using 5 mm steel beads at 20 Hz for 8 min (TissueLyser, QIAGEN, Hilden, Germany), and isolation of total RNA was done according to the manufacturer’s instruction. Subsequent RNA purification was performed using the RNeasy Mini Kit (QIAGEN) and genomic DNA digestion (DNase-Set, QIAGEN) according to the manual. Total RNA of cell cultures samples was isolated using the RNeasy Mini Kit only. The concentration of extracted RNA was measured using the Nanodrop ND-1000 Spectrophotometer (Thermo Fisher Scientific). RNA was reversely transcribed into cDNA applying the High Capacity cDNA Reverse Transcription Kit (Life Technologies) with random primers. Quantitative PCR was carried out on the ABI Prism 7900HT Sequence Detection System using the SYBR Green PCR Master Mix (all Life Technologies). After an initial denaturation at 95 °C for 10 min, the cDNA was amplified by 40 cycles of PCR (95 °C, 15 s; 60 °C, 60 s). The relative gene expression levels were normalized using β-actin gene and quantified by the 2^‑ΔΔCt^ method [21]. Primer sequences are summarised in Table 1.

**Table 1:** Primer sequences used for quantitative real-time PCR.

**Symbol**	**Gene ID**	**Forward primer**	**Reverse primer**
*ACTB*	60	CAGCCATGTACGTTGCTATCCAGG	AGGTCCAGACGCAGGATGGCATG
*CD36*	948	GTTGATTTGTGAATAAGAACCAGAGC	TGTTAAGCACCTGTTTCTTGCAA
*FABP4*	2167	GGTGGTGGAATGCGTCATG	CAACGTCCCTTGGCTTATGC
*LPL*	4023	ACAGAATTACTGGCCTCGATCC	CTGCATCATCAGGAGAAAGACG
*NR1H3* (*LXR*α)	10062	CACCTACATGCGTCGCAAGT	GACAGGACACACTCCTCCCG
*PDK4*	5166	CTGGACTTTGGTTCAGAAAATGC	CCTTCAGAATGTTGGCGAGTCT
*PLTP*	5360	GACACCGTGCCTGTGCG	GGTGGAAGCCACAGGATCCT
*PPARA*	5465	TTGCTGTGGAGATCGTCCTG	CCGGGTGGTTGCTCTGC
*PPARG*	5468	CATGGCAATTGAATGTCGTGTC	CCGGAAGAAACCCTTGCAT
*SCD*	6319	TGCCCACCTCTTCGGATATC	TAGTTGTGGAAGCCCTCACCC
*SLC27A4 (FATP4)*	10999	CCCCCTCTACCACTCAGCAG	TTCTTCCGAATCACCACCGT
*ACADM*	34	TAATTGGTGACGGAGCTGGTTT	CAACAGCACCAGCAGCTACTACA
*ACOX2*	8309	TGATCAAACAGACAATGGCTTCC	GCAAGACCTGTGCAAAGCG
*CPT 2*	1376	GAGTTTTGAAAATGGGATTGGAA	AACCAGGGTCCCGAAATGTAG
*ECH1*	1891	TGCTCAGAGAGTTTCAGATCAACC	CTGTCCATGTTGGGCAAGCT
*HMGCS2*	3158	CTAGCCTCCCGAAAGTGTGT	GGTGGGGAGAAATTCACCTT
*Acadl*	11363	AGCCTGGGGCTGGAAGTGACTTA	CACGGTTGGTGACGGCCACG
*Acadm*	11364	ACGGGGGAAAGGCCAACTGGTAT	AGGATCTGGGTTAGAACGTGCCA
*Acly*	104112	CAGCCAAGGCAATTTCAGAGC	CTCGACGTTTGATTAACTGGTCT
*Acox1*	11430	CAGCACTGGTCTCCGTCATG	CTCCGGACTACCATCCAAGATG
*Actb*	11461	TGTCCACCTTCCAGCAGATGT	AGCTCAGTAACAGTCCGCCTAGA
*Adipoq*	11450	AGGAAAGGAGAGCCTGGAGA	CGAATGGGTACATTGGGAAC
*Apoa2*	11807	GACTGCAGCACAGAATCGCAGCA	CCAGCAGTGCGACCATTGCGA
*Apoc3*	11814	CGTAGGTGCCATGCAGCCCC	CAGCTCGGGCAGATGCCAGG
*Ccl11*	20292	AGAGCTCCACAGCGCTTCTA	GGAAGTTGGGATGGAGCCTGG
*Ccl3*	20302	GCTCCCAGCCAGGTGTCATTTTCC	GGGGTTCCTCGCTGCCTCCA
*Ccl5*	20304	CTCACTGCAGCCGCCCTCTG	CCGAGCCATATGGTGAGGCAGG
*Ccr2*	12772	TCAGCTGCCTGCAAAGACCAGA	CGGTGTGGTGGCCCCTTCAT
*Ccr5*	12774	AGACTCTGGCTCTTGCAGGATGGA	GGCAGGAGCTGAGCCGCAAT
*Cd36*	12491	GCTTGCAACTGTCAGCACAT	GCCTTGCTGTAGCCAAGAAC
*Cd40*	1678	CGGCGTTCACTGTAAGGAGT	GTTTTCTGCCCCACCTGCTA
*Cd80*	1701	ATACGACTCGCAACCACACC	GAGGGTCTTCTGGGGGTTTT
*Cd86*	2539	ACTGTCAGTGATCGCCAACT	TAGGTTTCGGGTGACCTTGC
*Cpt 1a*	12894	TCTGCAGACTCGGTCACCACTCAAG	GGCTCAGGCGGAGATCGATGC
*Cpt 2*	12896	AAGCAGCGATGGGCCAG	GAGCTCAGGCAGGGTGACC
*Cxcl1*	14825	GGCCCCACTGCACCCAAACC	CAAGGCAAGCCTCGCGACCA
*Fabp4*	11770	CATGGCCAAGCCCAACAT	CGCCCAGTTTGAAGGAAATC
*Hsd11b1*	15483	CAGCCTCTGCTCACTACATTGC	AGTCCGCCCATGAGCTTTC
*Ifi30*	65972	GTCAGCTGTACCAGGGAACG	GTCTGGGCTTTGTGGGACAT
*Ifit3*	15959	GATTTCTGAACTGCTCAGCCC	ATTCCCGGTTGACCTCACTCA
*Il27ra*	50931	AGGCCAGGCTACTCACTACA	GGGTTTGACTGCTCACGTTC
*Irak4*	266632	ACAGCGACAACCTGTGCTTA	GTGTACCATCCAGGCAGGAC
*Irf5*	27056	CCCTGTCCCAGACCCAAATC	AGGTCCGTCAAAGGCAACAT
*Lgals3*	16854	TGGGGCCTACCCCAGTGCTC	GGCACCGTCAGTGGTCCAGC
*Nr1h3 (Lxrα)*	22259	GCTCTGCTCATTGCCATCAG	TGTTGCAGCCTCTCTACTTGGA
*Pck1*	18534	ATCATCTTTGGTGGCCGTAG	CATGGCTGCTCCTACAAACA
*Ppargc1a*	19017	TCCCATACACAACCGCAGTCGC	GGGGTCATTTGGTGACTCTGGGGT
*Ppargc1b*	170826	GGGAAAAGGCCATCGGTGAA	CAGCACCTGGCACTCTACAA
*Ptgs2*	19225	CCCTGCTGCCCGACACCTTC	CCAGCAACCCGGCCAGCAAT
*Tirap*	117149	CTTCATCCTCCTCCGTCCCA	TGCCTGAACCAGTCAGCTATC
*Tlr2*	24088	AACCTCAGACAAAGCGTCAA	TCCTGAGCAGAACAGCGTTT
*Tlr5*	53791	GAATCCCGCTTGGGAGAACA	TTCCAAGCGTAGGTGCTCTG
*Tlr6*	21899	AGTGCTGGAAATAGAGCTTGGA	TATTAAGGCCAGGGCGCAAA
*Tlr7*	170743	AAGAAAGATGTCCTTGGCTCCC	TCAAGAGGTCTGGTGGAGGA
*Tnf*	21926	AGCCCACGTCGTAGCAAACCA	CATGCCGTTGGCCAGGAGGG
*Tnfaip2*	3590	GATTCTCTGAACCGCCTGCT	TAAACAGCGGCTTCAGGTCC
*Tnfaip3*	4352	CCTGCCCAGGAGTGTTACAG	CGCGAAGTTCAGGTCCACT
*Tollip*	54473	GGATGACCGCATAGCTTGGA	CTGGGAGGGACGTGTAGGA
*Ucp1*	22227	CACGGGGACCTACAATGCTT	TAGGGGTCGTCCCTTTCCAA
*Ucp2*	22228	CGCCTTCTACAAGGGGTTCA	CGAGATTGGTAGGCAGCCAT
*Ucp3*	22229	ACAAAGGATTTGTGCCCTCC	TCAAAACGGAGATTCCCGCA

### Animals and diets

Animal studies were approved by the State Office of Health and Social Affairs (Berlin, Germany) and were carried out according internationally approved guidelines. All animals were singly housed under temperature-, humidity- and light-controlled conditions (22°C, 50% humidity, 12 h light/12 h dark cycle). Mice had *ad libitum* access to food and water. Mice, food and water were weighed in a regularly to determine changes in body weight and food and water intake. Low-fat diet (LFD, D12450B, 10 kcal% fat, 18.0 MJ/kg, ssniff, Soest, Germany) was composed as follows: 4.1% crude fat, 18.1% crude protein, 26.6% starch, 35.5% sugar/dextrines, 4.7% crude fibre. High-fat diet (HFD, D12492, 60 kcal% fat, 25.3 MJ/kg, ssniff) was composed as follows: 34.0% crude fat, 24.1% crude protein, 1.1% starch, 23.8% sugar/dextrines, 6.0% crude fibre. 

Two different mice studies were performed. For the prevention study, lean male 8 week-old C57BL/6 mice were weighed and distributed equally to three groups (*n*=14 each) and were fed over 20 weeks with either a LFD, HFD or HFD with 200 mg/kg/d CFE. Therefor, lyophilised CFE was diluted in drinking water (1.1-2.3 mg/ml depending on body weight). Blood was taken from the submandibular or tail vein of conscious mice for testing of blood parameters before dosing and after 6, 12, 17 and 20 weeks. After 20 weeks of dosing, fasted mice were killed by cervical dislocation and tissues were stored at -80 °C before use. 

For the therapy study, we subjected male 6-week old C57BL/6 mice to an HFD for 18 weeks to induce obesity and insulin resistance. Prior to compound treatment, mice were weighed and distributed uniformly to three groups (n=9-14 each). Mice were fed over 6 weeks with an HFD without compound (vehicle), with 4 mg/kg/d (34 µg/ml) rosiglitazone (RGZ) or with 200 mg/kg/d (2.3 mg/ml) CFE diluted in drinking water. After 2 weeks of treatment mice were subjected to an oral glucose tolerance test (OGTT). After 6 weeks of dosing, fasted mice were killed by cervical dislocation. Hematocrit was measured by weighting of blood samples before and after plasma separation and is presented as wt/wt %.

### Oral glucose tolerance test (OGTT)

Mice were fasted overnight before being subjected to an oral glucose (Sigma-Aldrich) dose of 2 g/kg body weight. Blood was taken from the tail vein at the indicated time points and blood glucose was analysed in a Hemocue B-Glucose analyser (Hemocue, Großostheim, Germany). Whole blood was further collected using Microvette lithium-heparin coated capillary tubes (CB300, Sarstedt, Nürnbrecht, Germany). After centrifugation for 5 min at 2000 *g* at 4°C, plasma was collected and stored at -80°C for subsequent measurements of insulin. 

### Metabolic parameters measurements

Whole blood glucose was analysed in a Hemocue B-Glucose analyser. Plasma glucose was measured using the Amplex Red Glucose Assay Kit (Life Technologies). Plasma triacylglycerol, NEFA, HDL and LDL/VLDL cholesterol and plasma alanine transaminase (ALT) were determined with colorimetric quantification kits (Biovision, Milpitas, CA, USA). Insulin (Insulin Ultrasensitive EIA, ALPCO, Salem, NH, USA), and osteocalcin (BGLAP bone gamma-carboxyglutamate (gla) protein, ABIN415574, antibodies-online, Aachen, Germany) were determined in plasma samples using ELISA. TNFα, triacylglycerol and NEFA in liver tissues were analysed as described recently [20]. All assays were performed according to the manufacturer’s instructions. 

Homeostasis model assessment of insulin resistance (HOMA-IR) was determined according to HOMA-IR = fasting blood glucose (mg/dl) × fasting insulin (µU/ml)/405.

### Statistical Analyses

Data are presented as mean ± standard error of mean (SEM) if not otherwise denoted. Statistical tests were performed in GraphPad Prism 5.0. For comparison of CFE- and vehicle-treated samples statistical significance was examined by unpaired Student’s t-test if not otherwise stated. A *p* value ≤ 0.05 was defined as statistically significant.

## Results

### CFE contains selective PPAR modulators (SPPARMs) and particularly activates PPARγ

**Figure 1:**
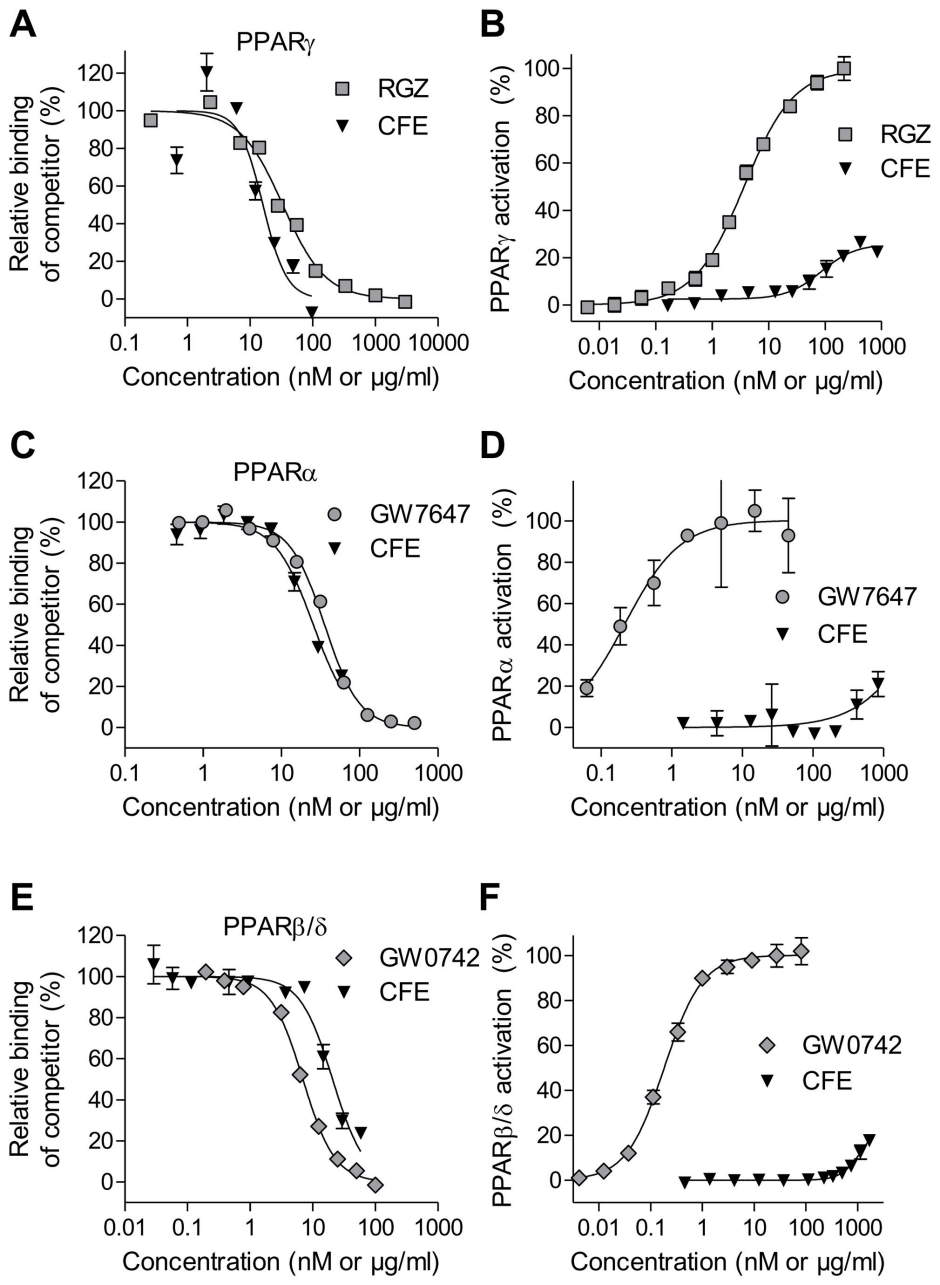
Binding and transcriptional activation of PPARs by natural-products contained in camomile flowers extract (CFE). (A, B) PPARγ bound and activated by CFE (µg/ml) or rosiglitazone (nM). (C, D) PPARα bound and activated by CFE (µg/ml) or GW7647 (nM). (E,F) PPARβ/δ bound by CFE (µg/ml) or GW0742 (nM). Binding of compounds was measured in a competitive time-resolved fluorescence resonance energy transfer assay. Transcriptional activation was determined in a reporter gene assay and is represented relative to the reference compound. Data are expressed as mean ± SD (n=3-4).

Initially, binding of PPARs by small molecules from the ethanolic chamomile flowers extract (CFE) was analyzed using a time-resolved fluorescence resonance energy transfer (TR-FRET)-based competitive binding assay. Titration of the PPARγ ligand-binding domain with CFE revealed an overall binding affinity constant (*K*_i_) of 6 µg/ml (Figure 1A and Table 2). In a reporter gene assay, CFE induced partial PPARγ activation with a half-maximal effective concentration (EC_50_) of 86 µg/ml and with a maximal potency of 26% (Figure 1B) compared to rosiglitazone (RGZ). CFE additionally contains natural products that bound and activated PPARα (K_i_ = 10 µg/ml, EC_50_ = 3750 µg/ml, Figure 1C,D) and PPARβ/δ (K_i_ = 9 µg/ml, EC_50_ = 1204 µg/ml Figure 1E, F and Table 2). 

**Table 2:** PPAR binding and activation data of chamomile flowers extract (CFE) and reference compounds.

**Receptor**	**Parameter**	**CFE**	**RGZ**	**GW7647**	**GW0742**
PPARα	K_i_ (µg/ml or nM)	10	n.d.	13	n.d.
	EC_50_ (µg/ml or nM)	^≈^ 3750	n.d.	0.2	n.d.
	Efficacy (%)	n.d.	n.d.	100	n.d.
PPARβ/δ	K_i_ (µg/ml or nM)	9	n.d.	n.d.	3
	EC_50_ (µg/ml or nM)	1204	n.d.	n.d.	0.2
	Efficacy (%)	n.d.	n.d.	n.d.	100
PPARγ	K_i_ (µg/ml or nM)	6	12	n.d.	n.d.
	EC_50_ (µg/ml or nM)	86	4	n.d.	n.d.
	Efficacy (%)	26	100	n.d.	n.d.

CFE is presented in µg/ml, RGZ, GW7647 and GW0742 are given in nM. CFE, camomile flowers extract (µg/ml). RGZ, rosiglitazone (nM). n.d, not determined.

These results suggested that the ethanolic extract from chamomile flowers contain selective PPAR modulators (SPPARMs), which particularly activate PPARγ and, additionally - but with about at least one order of magnitude lower half-maximal effective concentration (EC_50_) – the isotypes PPARα and β/δ. This extract may thus modulate gene expression in PPAR-expressing target cells.

### CFE activates PPARγ in human primary adipocytes

**Figure 2:**
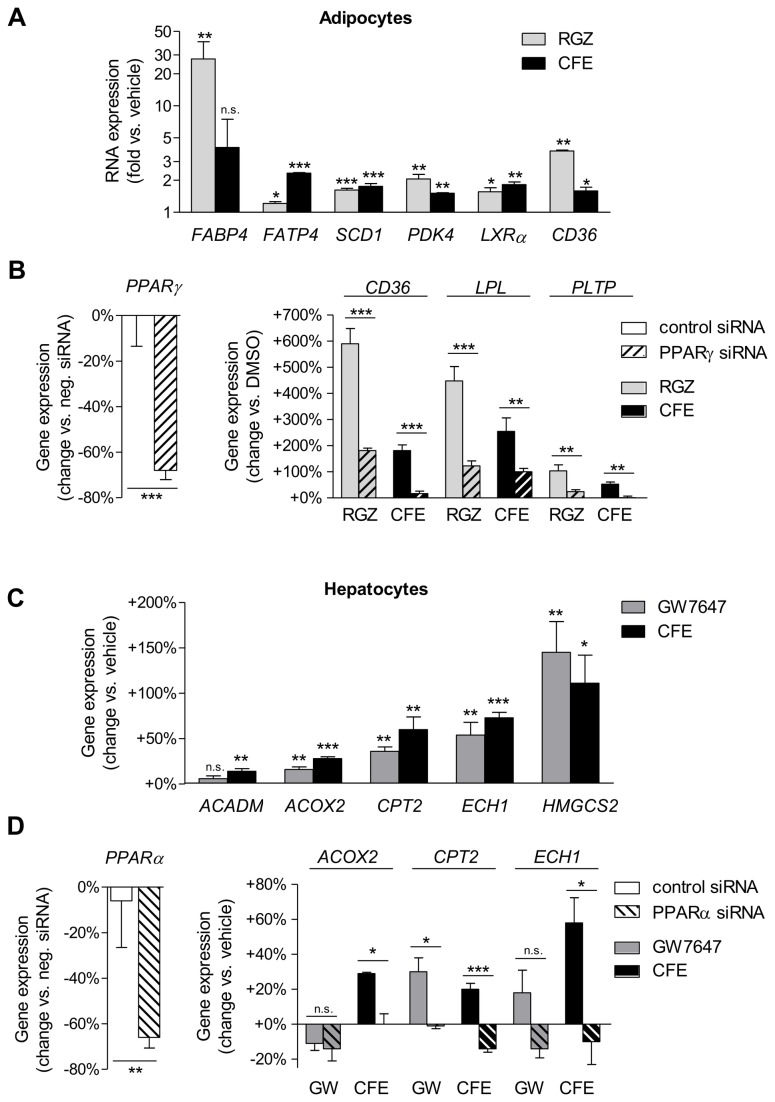
Gene expression profile of camomile flowers extract (CFE) in human adipocytes and hepatocytes. (A) Human primary adipocytes were treated with either 10 µM RGZ, 300 µg/ml CFE or vehicle only for 24 h. Gene expression was analyzed by qPCR. (B) Human primary adipocytes were transfected with PPARγ siRNA (hatched bars) or control siRNA (unhatched bars) and were treated with either 10 µM RGZ, 300 µg/ml CFE or vehicle only for 24 h. (C) Human HepG2 hepatocytes were treated with either 10 µM GW7647, 300 µg/ml CFE or vehicle only for 24 h and gene expression was analyzed by qPCR. (D) Human HepG2 hepatocytes were transfected with PPARα siRNA (hatched bars) or control siRNA (unhatched bars) and were treated with either 10 µM GW7647, 300 µg/ml CFE or vehicle only for 24 h. Data are expressed as mean ± SEM (*n*=2-4/group). n.s. not significant, *p≤0.05, **p≤0.01, ***p≤0.001 vs. vehicle. RGZ, rosiglitazone ; CFE, camomile flowers extract; GW, GW7647.

Activation of PPARγ, in particular in adipocytes, is a well-established strategy for antidiabetic treatment [22]. In accordance with partial PPARγ activation in the reporter gene assay, CFE-treated human primary adipocytes showed increased expression of PPARγ target genes such as the fatty acid binding protein 4 (*FABP4*), the fatty acid transport protein 4 (*FATP4*) or the liver X receptor alpha (*LXRa* or *NR1H3*) (Figure 2A). Whereas FABP4 expression is a marker of adipocyte differentiation and lipid accumulation [23], FATP4 facilitates fatty acid uptake by the adipocytes, and LXRα is a mediator of antiinflammatory gene expression [24].. Noteworthy, the increase in *FABP4* expression was 10-fold less with CFE as with RGZ (Figure 2A), indicating less induction of adipogenesis, a process that might be associated with body weight gain in vivo. Knockdown of PPARγ in these cells by siRNA led to a significant reduction of CFE-induced gene expression (Figure 2B), indicating specific modulation of PPARγ-derived transcription in adipocytes by CFE.

### CFE activates PPARα in human hepatocytes

Activation of PPARα that is in particular highly expressed in liver, is a well-established strategy for treating metabolic diseases including fatty liver, which can systemically also have beneficial effects on insulin sensitivity [25]. We thus determined expression of target genes of PPARα in HepG2 liver cells. Similarly to the PPARα agonist GW7647, CFE treatment significantly induced expression of genes responsible for fatty acid β-oxidation such as the acyl-CoA oxidase 2 (*ACOX2*), the carnitine palmitoyltransferase 2 (*CPT 2*) and the enoyl CoA hydratase 1 (ECH1), and of the ketogenic HMG-CoA synthase 2 (*HMGCS2*) gene (Figure 2C). Knockdown of PPARα in HepG2 cells diminished the CFE-induced gene expression (Figure 2D), indicating that in liver cells CFE mediates gene expression of PPARα target genes.

### CFE shows antidiabetic effects in insulin-resistant obese DIO mice

**Figure 3:**
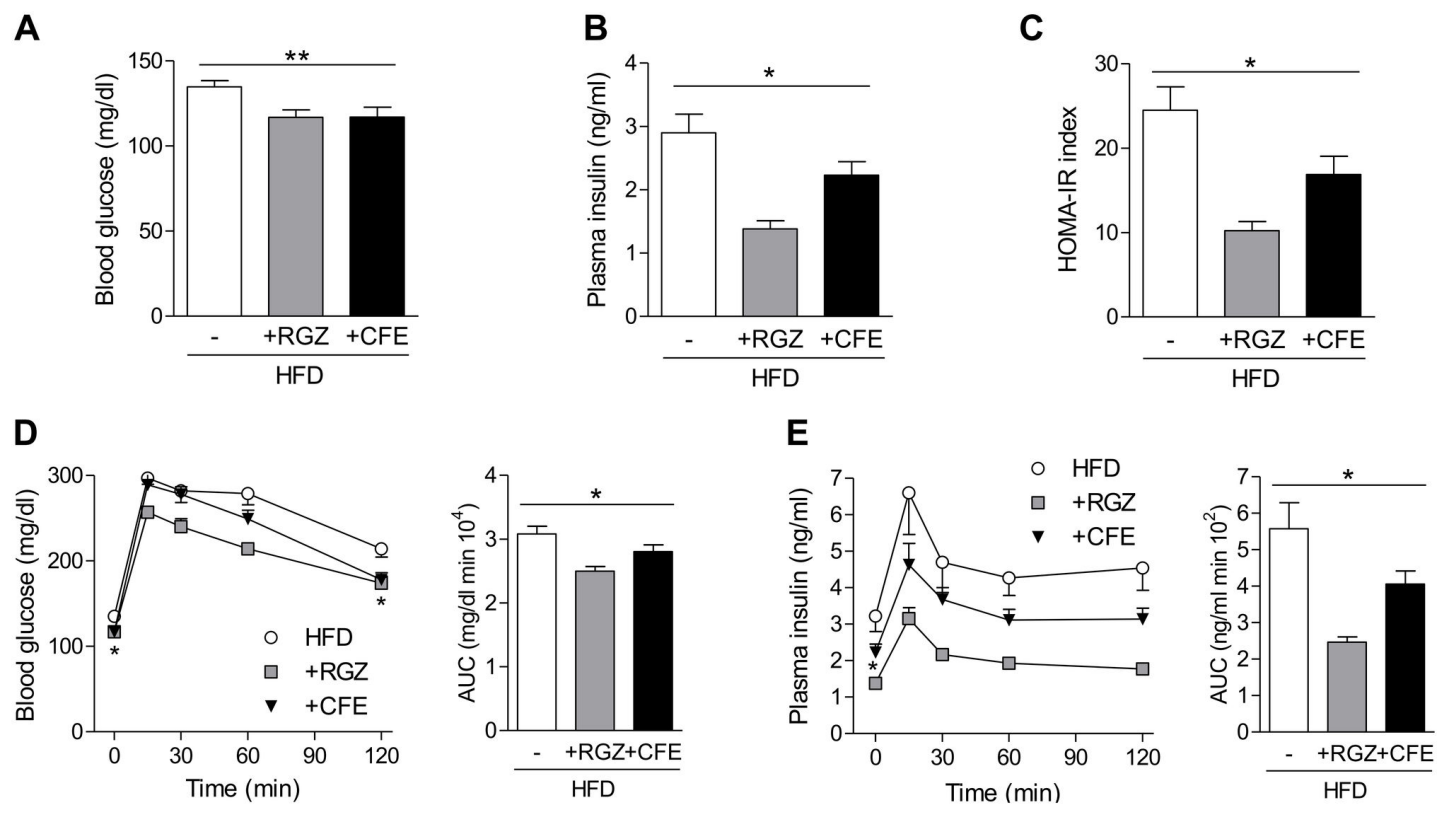
Antidiabetic effects of camomile flowers extract in insulin-resistent DIO mice. (A) Fasting blood glucose of untreated HFD-fed mice or mice treated for 2 weeks with RGZ or CFE. (B) Fasting plasma insulin after 2 weeks of treatment. (C) Effect of treatment for 2 weeks on insulin resistance determined by homeostatic model assessment of insulin resistance (HOMA-IR). (D, E) Glucose and insulin concentrations during oral glucose tolerance test (OGTT) after 2 weeks of treatment with vehicle, RGZ or CFE. AUC, area under the curve. Data are expressed as mean ± SEM. *p≤0.05, **p≤0.01 vs. vehicle-treated HFD-fed mice. HFD, high-fat diet; VEH, vehicle (*n*=13-14); RGZ, rosiglitazone (*n*=8-14) ; CFE, camomile flowers extract (*n*=12-14).

In general, activation of PPARγ in adipocytes can lead to strong antidiabetic effects in an in vivo context. To investigate these effects of CFE in a common mouse model of insulin resistance, we treated obese HFD-fed C57/BL6 mice for 6 weeks with 200 mg/kg/d CFE, 4 mg/kg/d rosiglitazone (RGZ) or vehicle only through the drinking water. After 2 weeks of dosage CFE-treated mice featured considerably reduced the fasting blood glucose (13% decrease vs. vehicle, *p*=0.007) equally to the established antidiabetic drug RGZ (Figure 3A). CFE furthermore reduced fasting plasma insulin levels (23% decrease, *p*=0.02, Figure 3B). Although primarily developed for human clinical data, homeostatic model assessment of insulin resistance (HOMA-IR) is a common approach for estimating the progress of insulin resistance also in murine models [26]. Thus, CFE decreased the HFD-induced insulin resistance by 36% (*p*=0.01, Figure 3C), which was half as potent as RGZ (67% decrease, Figure 3C).

We further investigated insulin resistance using oral glucose tolerance tests (OGTT) by kinetically following the metabolic clearance of a standard glucose load (2 g/kg). CFE-treated mice revealed increased glucose clearance (Figure 3D) and concomitantly reduced insulin release (Figure 3E) after 2 weeks of treatment. As observed for basal metabolic levels (Figures 3A-C), CFE alleviated HFD-induced insulin resistance half as effective as RGZ, indicating considerable antidiabetic effects of this plant extract.

To investigate the role of PPARγ activation in vivo, we analysed the expression of PPARγ target genes in the visceral white adipose tissue (WAT) of these mice (Figure S1). CFE activated transcription of important genes involved in lipid metabolism, such as the PPARγ coactivator 1 alpha and beta (*Ppargc1a* and *Ppargc1b*), the uncoupling protein 1 (*Ucp1*), the ATP citrate lyase (*Acly*) and the phosphoenolpyruvate carboxykinase 1 (*Pck1*), suggesting regulation of adipose tissue lipid metabolism similar to RGZ (Figure S1A). 

Type 2 diabetes seems to be causally linked to adipose tissue inflammation [27]. As shown in Figure S1B, we also measured in the WAT of treated mice the expression of a panel of HFD-induced genes involved in inflammation [20,28]. However, gene expression of inflammatory markers remained largely unchanged with either RGZ or CFE treatment. 

### CFE considerably improves dyslipidemia in obese DIO mice

**Figure 4:**
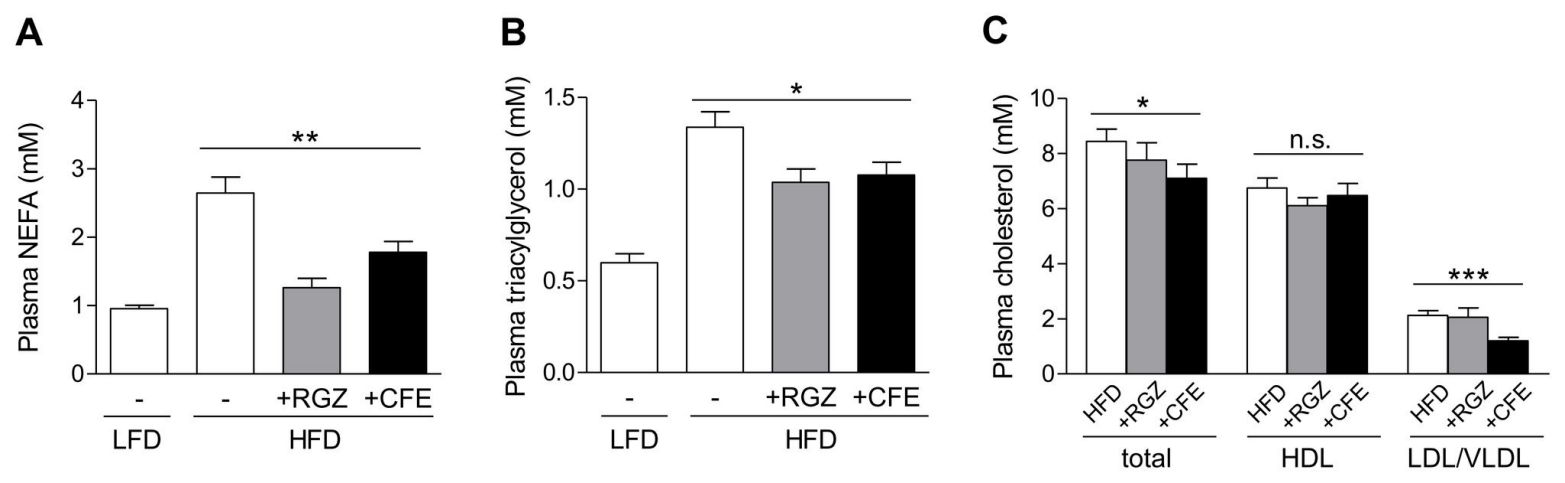
Camomile flowers extract improves dyslipidemia in obese DIO mice. (A) Fasting plasma NEFA after 6 weeks of treatment. (B) Fasting plasma triacylglycerol after 6 weeks of treatment. (C) Fasting plasma total, HDL and LDL/VLDL cholesterol in DIO mice after 6 weeks of treatment. Data are expressed as mean ± SEM. *p≤0.05, **p≤0.01, ***p≤0.001, n.s. not significant vs. vehicle-treated HFD-fed mice. LFD, low-fat diet; HFD, high-fat diet; VEH, vehicle (*n*=13-14); RGZ, rosiglitazone (*n*=8-14) ; CFE, camomile flowers extract (*n*=12-14).

Dyslipidemia is another important feature of metabolic diseases, which can be inhibited by activation of PPARs [29]. During 6 weeks CFE strongly decreased the HFD-induced rise in the fasting plasma concentrations of non-esterified fatty acids (NEFA) by 53% (Figure 4A) and further reduced the plasma triacylglycerol gain by 35% (Figure 4B) comparable to the strong synthetic PPARγ agonist RGZ. In contrast to RGZ, CFE-fed mice also showed reduced plasma levels of total cholesterol (16% decrease) and LDL/VLDL cholesterol (43% decrease). Thus, additional amelioration of HFD-induced hypercholesterolemia by the CFE might be achieved, at least in part, through activation of PPARα (Figure 4C). 

### CFE prevents insulin resistance, non-alcoholic fatty liver disease (NAFLD) and hepatic inflammation in long-term treated HFD-fed mice

**Figure 5:**
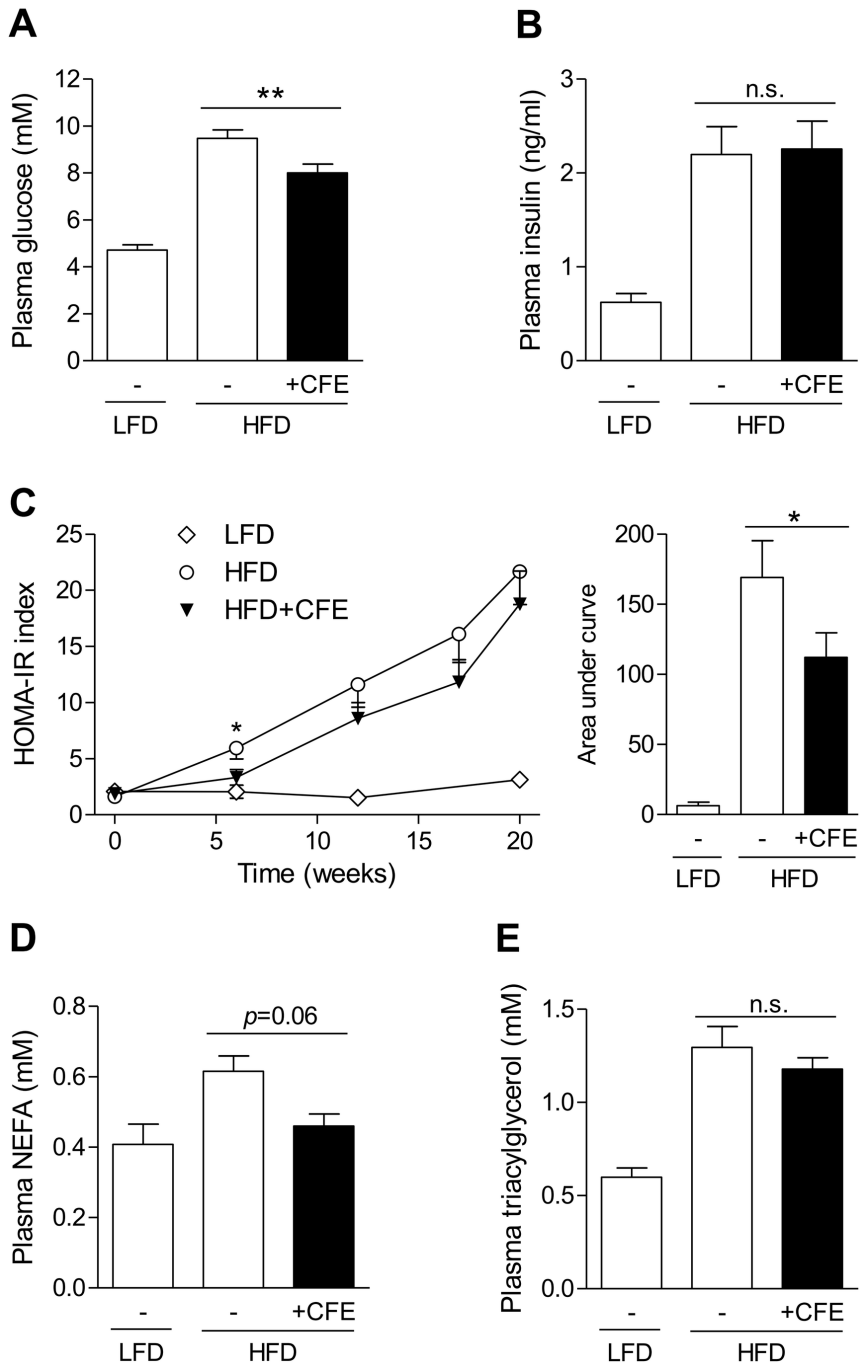
Prevention of insulin resistance and dyslipidemia by camomile flowers extract during HFD-feeding of healthy C57BL/6 mice. (A) Fasting plasma glucose of LFD-fed, untreated HFD-fed or HFD-fed mice treated for 20 weeks with CFE (*n*=14/group). (B) Fasting plasma insulin after 20 weeks of intervention (LFD *n*=7, HFD *n*=14, HFD+CFE *n*=14). (C) Effect of 20 weeks preventive feeding on homeostatic model assessment of insulin resistance (HOMA-IR) index (LFD *n*=7, HFD *n*=13, HFD+CFE *n*=12). AUC, area under the curve. (D) Fasting plasma NEFA after 20 weeks of treatment (LFD *n*=8, HFD *n*=10, HFD+CFE *n*=9). (E) Fasting plasma triacylglycerol after 20 weeks of treatment (LFD *n*=8, HFD *n*=14, HFD+CFE *n*=14). Data are expressed as mean ± SEM. n.s. not significant, *p≤0.05, **p≤0.01 vs. untreated HFD-fed mice. LFD, low-fat diet; HFD, high-fat diet; CFE, camomile flowers extract.

To test potential effects of preventive administration of CFE on metabolic disorders we fed healthy C57BL/6 mice for 20 weeks with either a LFD, a HFD or a HFD with simultaneous CFE administration (200 mg/kg/d). Similar to the therapeutic study, CFE inhibited the HFD-induced rise in plasma glucose and the associated formation of insulin resistance by 35%, as assessed by HOMA-IR (Figures 5A-C). Furthermore, CFE partly stopped the increase in plasma NEFA and triacylglycerols (Figures 5D-E), corroborating the above described effects observed in short-term treated DIO mice (Figures 3-4).

**Figure 6:**
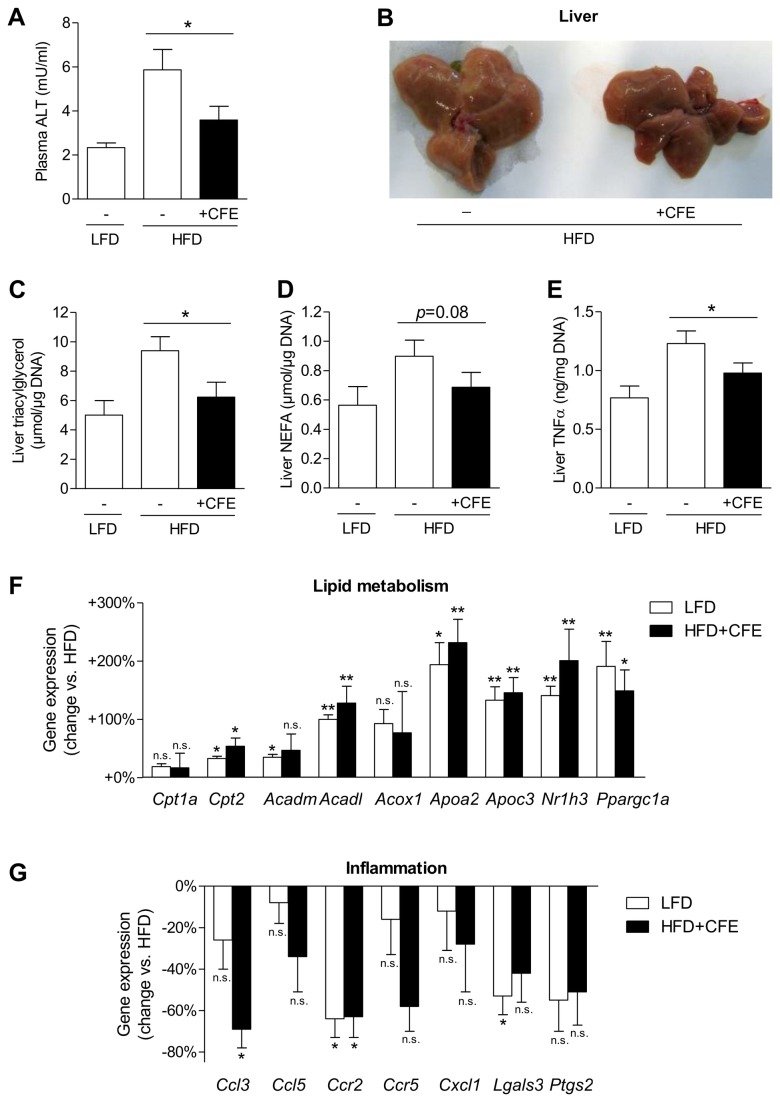
Prevention of non-alcoholic fatty liver disease (NAFLD) by camomile flowers extract during HFD-feeding of healthy C57BL/6 mice. (A) Plasma levels of liver alanine transaminase (ALT) after 20 weeks of treatment (*n*=12/group). (B) Effect treatment over 20 weeks on liver morphology. (C, D) Liver concentrations of triacylglycerol and NEFA after 20 weeks of treatment (*n*=10-12/group). (E) TNFα protein concentrations in liver (*n*=9/group). (F) Liver qPCR analysis of genes involved in lipid metabolism (*n*=4-6 pools, 2 mice/pool). (G) Liver qPCR analysis of genes involved in inflammation and macrophage infiltration (*n*=4-6 pools, 2 mice/pool). Data are expressed as mean ± SEM. n.s. not significant, *p≤0.05, **p≤0.01 vs. untreated HFD-fed mice. LFD, low-fat diet; HFD, high-fat diet; CFE, camomile flowers extract.

Moreover, HFD-feeding over 20 weeks resulted in considerably elevated plasma levels of alanine transaminase (ALT), indicating liver damage due to steatohepatitis. CFE-treatment strikingly prevented this raise in plasma ALT levels by 64% (*p*=0.05, Figure 6A). Examination of the liver tissues clearly showed reduced formation of fatty liver of CFE-treated mice (Figure 6B), which was accompanied by reduced liver triacylglycerols (72% decrease, Figure 6C) and liver NEFA concentrations (64% decrease, Figure 6D). Consistently, HFD-feeding resulted in a boost of protein expression of pro-inflammatory tumor necrosis factor alpha (TNFα) that was significantly prevented by application of CFE by 54% (Figure 6E). Analyses of liver gene expression consistently showed induction of fatty acid oxidation, e.g. through Cpt *2*, medium and long-chain acyl-Coenzyme A dehydrogenase (*Acadm* and *Acadl*), and *Ppargc1a* (Figure 6F). Furthermore, CFE completely prevented the HFD-induced raise of inflammatory transcripts in the liver (Figure 6G). These results indicate that early administration of CFE effectively protects from HFD-induced fatty liver disease and associated liver inflammation.

### CFE does not induce the adverse side-effects associated with PPARγ-activating thiazolidinediones

**Figure 7:**
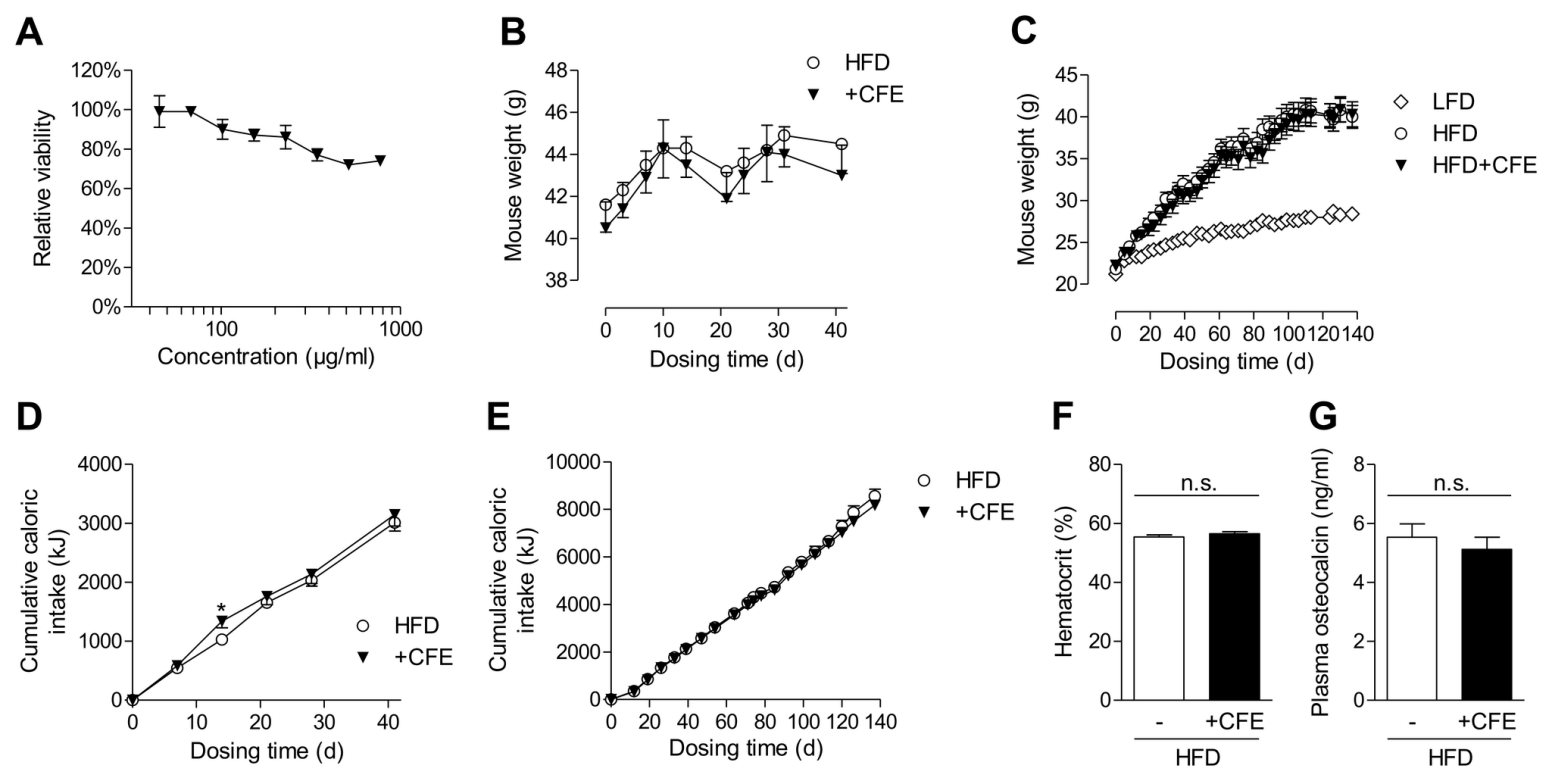
Camomile flowers extract (CFE) does not induce adverse effects commonly linked with PPAR agonists. (A) Effect of CFE on cellular viability in human HepG2 cells after treatment for 24 h. Data are expressed as mean ± SD (n=3/group). (B, C) Mouse body weight during treatment of DIO mice for 6 weeks with CFE or HFD alone (B) and during the preventive study by 20 weeks feeding of healthy C57BL/6 mice with LFD, HFD alone or HFD with CFE (C). Data are expressed as mean ± SEM (*n*=14/group). Data are shown as mean ± SEM. (D, E) Food intake during treatment of DIO mice for 6 weeks with CFE or HFD alone (D) and during the preventive study by 20 weeks feeding of healthy C57BL/6 mice with LFD, HFD alone or HFD with CFE (E). Data are expressed as mean ± SEM (*n*=14/group). (F) Hematocrit of treated DIO mice after 6 weeks (mean ± SEM, n=14/group). (G) Effect of CFE on plasma osteocalcin levels after treatment of DIO mice for 6 weeks (mean ± SEM, n=14/group). *p≤0.05, n.s. not significant vs. untreated HFD-fed mice. LFD, low-fat diet; HFD, high-fat diet; CFE, camomile flowers extract.

CFE has been used as a safe extract for various therapeutic applications [30]. Consistently, in vitro viability assays did not indicate significant toxicity effects up to 800 µg/ml CFE (Figure 7A). Adverse body weight gain is a frequent side effect of PPARγ activation by rosiglitazone. However, CFE-treated mice did not show any body weight changes compared to untreated mice, neither during treatment for 6 weeks (Figure 7B) nor during long-term treatment (Figure 7C). At the same time, CFE treatment did not change food consumption of the mice (Figure 7D and E). CFE treatment did also not impair hematocrit levels (an indicator of fluid retention and potential cardiovascular complications) in DIO mice (Figure 7F), however, in our hands RGZ-treated mice did not show any signs of fluid retention after 6 weeks of treatment as we have shown recently [31]. Another well-known side-effect of synthetic PPARγ agonists is the impairment of osteoblastogenesis leading to osteoporosis and increased fracture risk. In HFD-fed mice, CFE-treated DIO mice did not show any change in plasma bone osteocalcin levels (Figure 7G), indicating no adverse effects on bone cell turnover with CFE, contrary to RGZ-treated mice [31]. These various results suggest that administration of chamomile flowers extracts does not induce the typical side effects of highly efficient synthetic PPARγ agonists.

## Discussion

Chamomile is a popular herb that has been used for thousands of years in ancient Egypt, Greece, and Rome [30]. Its phytomedicinal application is mainly predicated on its antioxidant, antiinflammatory, antiseptic, antispasmodic and wound-healing effects [32], especially in gastrointestinal disorders [19,33]. To our knowledge, only few studies showed antidiabetic effects of diverse, mainly water chamomile extracts, and the underlying mechanisms of action are still poorly understood. Furthermore, there are so far no studies on the effects of chamomile flowers extracts in diet-induced obesity (DIO) mice, one of the most often applied standard models for the formation of insulin resistance.

Cemek et al. investigated the effects of an ethanolic extract of *Matricaria chamomilla* in streptozotocin (STZ)-induced diabetic rats, and observed significant reductions in blood glucose after 7 to 14 days with 50-100 mg/kg/d extract administered orally [34]. Kato et al. [35] tested instead of ethanolic, hot water chamomile extracts and major constituents in STZ-induced diabetic rats, and they observed decreased hyperglycaemia with 500 mg/kg/d orally applied extract. Eddouks et al. reported that aqueous extracts of the taxonomically related *Chamaemelum nobile* reduced blood glucose levels after 2 weeks of 20 mg/kg/d extract dosing in normal and STZ-induced rats [36]. Additionally, Gupta et al. could observe hepatoprotective effects with chamomile extracts in Paracetamol-intoxicated rats [37]. However, all these studies could not sufficiently shed light on the underlying molecular mechanisms of the observed physiological effects. 

Due to their nature as nutrient-sensors the PPARs are interesting targets for dietary intervention and treatment of metabolic disorders with plant-derived mixtures of natural products. In this study, we focused on the antidiabetic and hypolipidemic effects and the interaction of ethanolic chamomile flowers extract (CFE) with two important subtypes of the peroxisome proliferator-activated receptors, namely PPARγ and PPARα. Activation of PPARγ, a key ligand-dependent transcriptional regulator of adipocytes, is a well-known approach for systemically improving insulin sensitivity, whereas activation of PPARα in the liver is efficient to counteract metabolic disorders of this organ and indirectly alleviates a number of other complex disorders including plasma cholesterol levels or insulin resistance [29]. PPARβ/δ is much more ubiquitously expressed in many diverse cells than the other two PPAR subtypes, and its potential impact in a cellular or physiological context is consequently difficult to pinpoint. PPAR-activating molecules such as the thiazolidinediones (PPARγ agonists) and the fibrates (PPARα agonists) have been widely applied as drugs since the 1990s and 1960s, respectively [38]. More recently, ligands with partial instead of full PPAR activation (SPPARMs) have been proposed to exert strong metabolic effects with potentially fewer side effects [14,39,40]. 

Consequently, dosing of high-fed diet (HFD)-fed obese mice with CFE alleviated insulin resistance approximately half as effective as RGZ, and further strongly reduced plasma NEFA and triacylglycerol similar to RGZ. Additionally, CFE decreased hypercholesterolemia and prevented the development of non-alcoholic fatty liver disease (NAFLD) and hepatic inflammation. These effects of CFE were not observed with the specific PPARγ activator RGZ, suggesting additional effects from activating PPARs in liver. The observed hypocholesterolemic and hepatoprotective combined with antidiabetic effects are shared with other PPAR pan agonists such as the fibrates or the amorfrutins, suggesting similar molecular mechanisms based on synergistic activation of PPARγ and PPARα [20,31]. 

Future in-depth analyses are needed to better understand the complex beneficial effects of the CFE. Such studies may include the role of transcriptional control of all three PPAR subtypes or studying additional peripheral metabolic target tissues. Without doubt, the pathophysiology of metabolic diseases as well as the pharmacological intervention thereof are based on complicated networks of metabolic events (carbohydrate/lipid digestion and uptake, storage, distribution, breakdown, neogenesis, excretion) and diverse tissues (e.g. pancreas, adipose, liver, skeletal muscle, macrophages) [28], and modulation of PPARs by natural products in plant extracts is only one aspect of the underlying mechanism(s) of action. Other possible targets of chamomile extracts might also be involved in the observed antidiabetic effects. For instance, Kato et al. [35] tested hot water chamomile extracts and major constituents *in vitro* on α-Amylase, intestinal α-Glucosidase, hepatic glycogen phosphorylase, aldose reductase and sorbitol dehydrogenase activity, which all have important roles in carbohydrate absorption or metabolism. These authors observed IC50 values ranging from 17 µg/ml to 5200 µg/ml, which is partly in the range of the affinity binding constants and EC_50_ values that we observed here for the PPARs. The complex CFE plant extract may thus contain many active ingredients addressing multiple targets relevant for the metabolic syndrome. 

Extracts of chamomile flowers contain a vast amount of detectable chemical constituents, and more than 100 different natural products have been identified so far, including flavonoids, sesquiterpenes and their derivatives, monoterpenes, coumarins and phenolic acids [19,30,35,41]. Many of these small molecules, predominantly the flavonoids, have already been identified as PPAR ligands, including genistein [42], daidzein [42], biochanin A [42,43], formononetin [42], glycitein [42], apigenin [43,44], chrysin [44], kaempferol [44] [43], quercetin [43,45] luteolin [43,46], diosmetin [43], and naringenin [43,47]. As in chamomile flowers the flavonoid class comprises more than 60% of the secondary phytochemical compounds [19], it seems futile to search for *the* specific PPAR agonist. It is rather probably the synergistic action of many flavonoids and other small molecules that mediate activation of nutrient-sensing PPARs and the antidiabetic, antiinflammatory and antihyperlipidemic effects of chamomile flowers extracts observed in this study. Besides PPARγ and its PPAR subtypes, other biological targets might be involved in the metabolic improvements observed in our mice studies. For instance, esculetin and quercetin, two major constituents of CFE, inhibit intestinal α-Glucosidases activities and reduce blood glucose in STZ-diabetic rats [35]. Luteolin and quercetin were shown to inhibit hepatic glycogen phosphorylase and increase liver glycogen content [35]. Chlorogenic acid, a phenolic acid present in chamomile flowers, may slow down carbohydrate absorption by inhibiting intestinal glucose transport [48]. Additional important effects of polyphenols comprise antioxidant activity, attenuating endoplasmic reticulum stress and repressing proinflammatory pathways [49]. 

**Figure 8:**
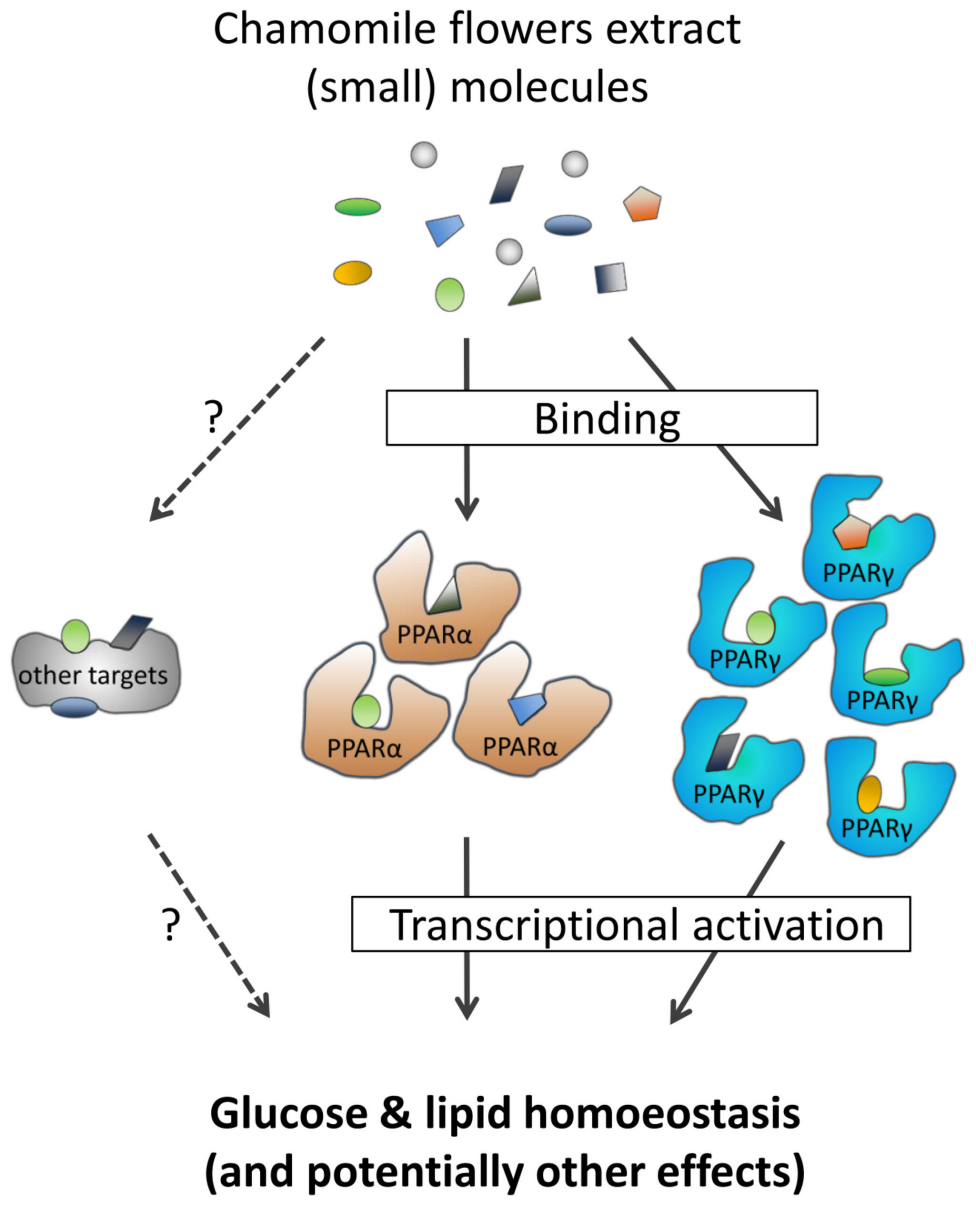
Concept of PPAR polypharmacology. Ethanolic extracts of chamomile flowers represent a complex mixture of plenty diverse compounds, particularly small molecules. In dependance of their bioavailability, a set of small molecules bind to peroxisome proliferator-activated receptors (PPARs) and potentially other yet unidentified targets. Ligand-binding then induces conformational changes in PPARs that lead to their transcriptional activation/modulation and thus to beneficial regulation of glucose and lipid metabolism. In summary, the polypharmacological mechanism is driven by the composition of structurally diverse small molecules in chamomile flowers extracts and by the large binding pocket of PPARs leading to promiscuous ligand-binding properties. The proportion of activation between the different PPAR subtypes is determined by the cellular context (e.g. cell type) and by the definite chemical composition of the plant extract.

As shown here, CFE seems to contain a set of numerous unidentified, more or less potent PPAR ligands of different chemical structure. The large binding pocket of the PPARs allows for promiscuous binding of ligands with large structural diversity [4]. We suggest that CFE allows for polypharmacological, synergistic modulation of PPARs and potentially a number of so far unexplored targets to influence various metabolic pathways, resulting in the beneficial physiological effects observed in this study (Figure 8). The multidrug/multitarget principle applied in this study is very difficult to pinpoint mechanistically in all details. But on the other hand this rather holistic approach may to some degree fulfill much better the polyetiological background of the metabolic syndrome than the reductionist approach of selective single-drug/single-target interventions, in particular for developing efficient preventive strategies to counteract age-associated metabolic diseases. Holistic approaches that make use of edible traditional plant extracts or active fractions thereof may effectively address early on dysregulated biological pathways, and minimize the risk of adverse side-effects. Furthermore, from a technical point of view, plant extracts do not require expensive isolation and enrichment of single compounds from complex mixtures or sophisticated chemical syntheses of active small molecules. Clearly, cultivation conditions and standardization of extract preparation are important but technically feasible issues.

In our mice studies the extract dose was 200 mg/kg per day, dissolved in drinking water (1-2 mg/ml) administered evenly throughout the day. For a human equivalent dose of 20 mg/kg/d and mean body weight of 80 kg the daily dose would be approximately 1600 mg extract. This amount could be reached for example with 2 to 4 cups of tea preparations (2-4 mg/ml) daily, which is a reasonable amount for dietary intervention. Clearly, solvent and extraction conditions will influence composition and stability of the extract. Time of intake could be a critical point, since chamomile extracts might inhibit intestinal carbohydrate uptake and transport [35] that is crucial particularly during or immediately after meal consumption. Furthermore, the circadian changes of nuclear receptors activation may influence the efficiency of the ingredients of the CFE [50]. In general, CFE is a safe and widely used extract that can be readily applied for humans. Future clinical studies may validate the observed antidiabetic and antihyperlipidemic effects of CFE to provide the basis for the development of efficient functional foods or nutraceuticals.

As demonstrated here, the application of plant-derived extracts as selective gene expression activators highlights the unique biological properties of natural resources to counteract metabolic diseases, and suggests that tailored dietary intervention may represent a fruitful approach for alleviating common diseases. 

## Supporting Information

Figure S1 **Gene expression in white adipose tissue of camomile flowers extract-treated DIO mice.** Visceral white adipose tissue of insulin-resistent DIO mice treated for 6 weeks with either HFD, HFD+RGZ or HFD+CFE was analysed by qPCR and is presented relative to untreated HFD-fed mice. (A) Genes involved in lipid metabolism. (B) Genes involved in inflammation and macrophage infiltration. Data are expressed as mean ± SEM (*n*=4-6 pools, 2 mice/pool). n.s. not significant, *p≤0.05, **p≤0.01 vs. untreated HFD-fed mice.
(TIF)
