# Supplementary figures and images for: Antidiabetic Effects of Chamomile Flowers Extract in Obese Mice through Transcriptional Stimulation of Nutrient Sensors of the Peroxisome Proliferator-Activated Receptor (PPAR) Family
Source: PLoS One. 2013 Nov 12;8(11):e80335. doi: 10.1371/journal.pone.0080335 (PMC3827197; doi:10.1371/journal.pone.0080335)

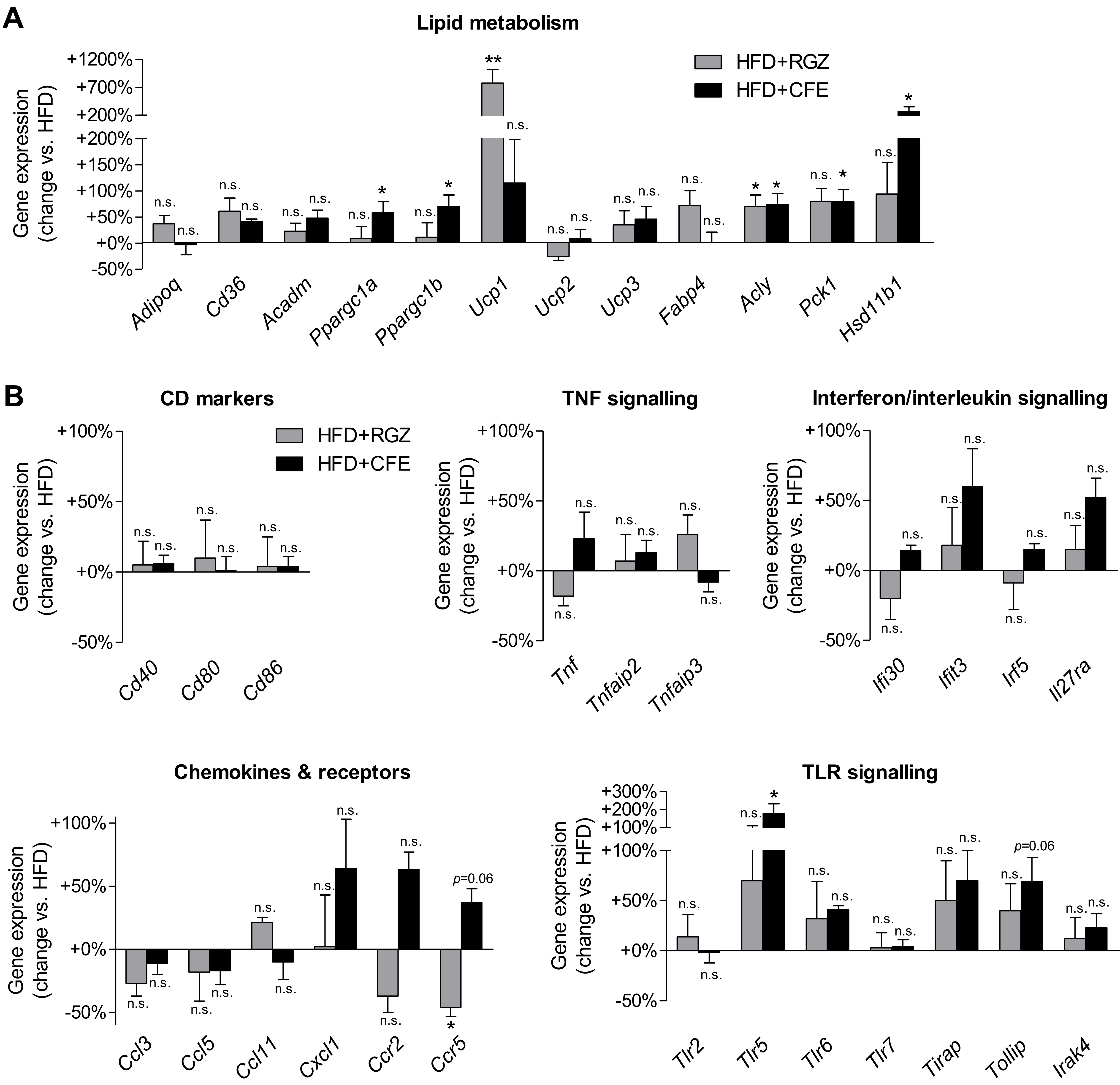

Supplement: Figure S1 — Gene expression in white adipose tissue of camomile flowers extract-treated DIO mice. Visceral white adipose tissue of insulin-resistent DIO mice treated for 6 weeks with either HFD, HFD+RGZ or HFD+CFE was analysed by qPCR and is presented relative to untreated HFD-fed mice. (A) Genes involved in lipid metabolism. (B) Genes involved in inflammation and macrophage infiltration. Data are expressed as mean ± SEM (n=4-6 pools, 2 mice/pool). n.s. not significant, *p≤0.05, **p≤0.01 vs. untreated HFD-fed mice. (TIF) [file pone.0080335.s001.tif]
